# Regulation of Mouse NK Cell Development and Function by Cytokines

**DOI:** 10.3389/fimmu.2013.00450

**Published:** 2013-12-12

**Authors:** Antoine Marçais, Sébastien Viel, Morgan Grau, Thomas Henry, Jacqueline Marvel, Thierry Walzer

**Affiliations:** ^1^CIRI, International Center for Infectiology Research, Université de Lyon, Lyon, France; ^2^U1111, INSERM, Lyon, France; ^3^Ecole Normale Supérieure de Lyon, Lyon, France; ^4^Centre International de Recherche en Infectiologie, Université Lyon 1, Lyon, France; ^5^UMR5308, CNRS, Lyon, France; ^6^Laboratoire d’Immunologie, Hospices Civils de Lyon, Centre Hospitalier Lyon Sud, Lyon, France

**Keywords:** natural killer cells, cytotoxicity, interferons, signal transduction, interleukin-15, interleukin-12, interleukin-18, TGF-beta

## Abstract

Natural Killer (NK) cells are innate lymphocytes with an important role in the early defense against intracellular pathogens and against tumors. Like other immune cells, almost every aspects of their biology are regulated by cytokines. Interleukin (IL)-15 is pivotal for their development, homeostasis, and activation. Moreover, numerous other activating or inhibitory cytokines such as IL-2, IL-4, IL-7, IL-10, IL-12, IL-18, IL-21, Transforming growth factor-β (TGFβ) and type I interferons regulate their activation and their effector functions at different stages of the immune response. In this review we summarize the current understanding on the effect of these different cytokines on NK cell development, homeostasis, and functions during steady-state or upon infection by different pathogens. We try to delineate the cellular sources of these cytokines, the intracellular pathways they trigger and the transcription factors they regulate. We describe the known synergies or antagonisms between different cytokines and highlight outstanding questions in this field of investigation. Finally, we discuss how a better knowledge of cytokine action on NK cells could help improve strategies to manipulate NK cells in different clinical situations.

Natural killer (NK) cells are Innate Lymphoid Cells (ILC) involved in the immuno-surveillance of cancers and in the early control of infections by intracellular pathogens (1). They can kill cells recognized as targets through a battery of surface receptors (2) and produce large amounts of IFN-γ upon activation (1). Recently, the growing ILC family has been reclassified into three groups according to the pattern of cytokine they secrete. In this classification, NK cells are part of the group 1 ILC subset (3). In mice, NK cells mainly develop in the bone marrow (BM) (4, 5). If the earliest committed NK cell progenitor (pre-pro NK) does not express CD122 (6) which is the β subunit of the IL-2/IL-15 receptor, expression of this molecule is acquired soon after at the NK precursor (NKP) stage (7). The expression of this receptor is thereafter conserved and is a hallmark of the NK cell population. This underlines the fact that the various aspects of NK cell development, homeostasis, and function are conditioned by IL-15. If this cytokine is fundamental, a variety of other cytokines have been shown to influence the behavior of NK cells, alone or in synergy. In this review, we aim to describe the complex interplay between the molecular pathways triggered by these cytokines. We restricted our field of investigation to the direct effects of cytokines on NK cells. We believe that a good understanding of these pathways is essential to the rational design of drugs targeting the various aspects of NK cell functions.

## The γc Family of Cytokines

**Figure 1:**
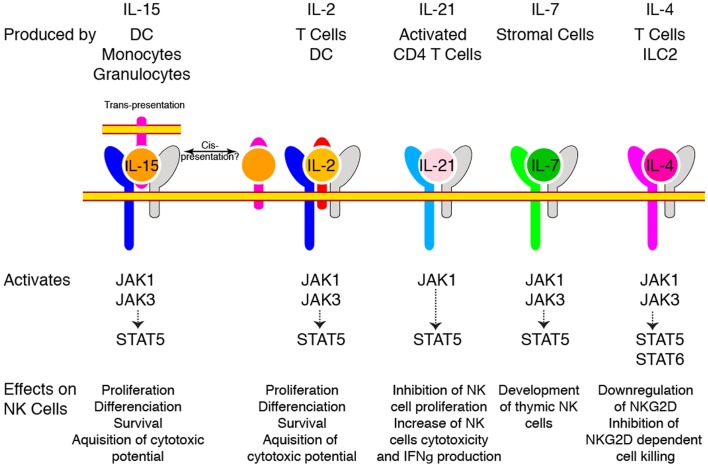
**γc Cytokines regulate NK cell homeostasis and function**. Different cytokines use γc as a co-receptor to regulate NK cell biology. The cellular source of these cytokines is indicated on the top. The different receptor chains and the major signaling pathways they trigger are shown. The effect of each cytokine on NK cells is also mentioned at the bottom.

The central role of cytokines sharing the γc subunit as a co-receptor (IL-2, IL-4, IL-7, IL-9, IL-15, and IL-21) in the constitution of the NK cell pool was appreciated some 20 years ago. Indeed, X-linked severe combined immunodeficiency patients presenting mutations leading to loss of γc function (8) and mice with targeted γc deletion (9) both presented a quasi absence of NK cells indicating that these cells relied on one or more of these cytokines. Absence of mature NK cells in mice genetically deficient in IL-2/15Rβ chain restricted the list of candidates to IL-2 and IL-15 (10). Finally, genetic ablation of IL-15 (11) or IL-15Rα (12), leading to a very similar phenotype with a near complete absence of mature NK cells, formally demonstrated the paramount importance of this cytokine for the generation of NK cells. In contrast, mice deficient for IL-2, IL-4, and IL-7 had normal NK cell numbers under homeostatic conditions (13). Importantly and despite expression of IL-2/15Rβ, NKPs appeared to be independent of γc signaling since they were present in normal numbers in γc deficient animals, suggesting that IL-15 only becomes important for subsequent maturation steps (13). Other γc cytokines are also involved in NK cell homeostasis and activation as summarized in Figure 1.

## IL-15

### Discovery/receptors/trans-presentation

IL-15 was discovered as a result of its “IL-2-like” stimulatory activity since it was able to support proliferation of IL-2 dependent cell lines (14, 15). Structurally, IL-15 is a 14–15 kDa protein presenting sparse sequence similarity (19%) but extensive 3-dimensional analogy with IL-2 and belonging to the same four α-helix bundle cytokine family (15). IL-15 and IL-2 both interact with receptor complexes containing the common gamma chain (γc) (16) and IL-2/15Rβ chain (15, 17). The γc subunit also takes part in the formation of the receptors for IL-4, IL-7, IL-9, and IL-21 (18) whereas IL-2/15Rβ is only used for signaling by IL-2 and IL-15. IL-2 and IL-15 receptors only differ by their α chain, IL-2Rα (CD25) being dedicated to IL-2 and IL-15Rα to IL-15 (19). In contrast to IL-2Rα, IL-15Rα alone displays a high affinity of binding for IL-15 (1.4 × 10^−11^M), equivalent to that of the heterotrimeric IL-2R for IL-2 (19). This last property is fundamental to understand the physiology of IL-15. Indeed, this high affinity, coupled to the fact that IL-15 and IL-15Rα are co-expressed by the same cells, allows intracellular binding of IL-15 to IL-15Rα in the endoplasmic reticulum. The complex is then shuttled to the cell membrane and presented to activate neighboring cells expressing IL-2/15Rβ/γc. This mechanism was called trans-presentation (20, 21) and proposed to explain the counter-intuitive fact that expression of IL-15Rα was not needed on responding NK cells to maintain their homeostasis, as would have been expected for a classical cytokine response scheme, but on neighboring cells (22–26). We can hypothesize that such a mechanism allows a very precise delivery of the cytokine stimulus perhaps coupled to other stimulating molecules. As NK cells rely on IL-15 for different purposes, trans-presentation would confine IL-15 signal to selective niches where it would sustain needs of specific NK cell sub-populations. Moreover, systemic availability of this cytokine could be detrimental as exemplified by the development of fatal leukemia in IL-15 transgenic animals (27, 28). The debate whether cis-presentation (i.e., autocrine presentation of IL-15) also occurs was recently revived by a paper showing that following bacterial challenge, NK cells produce and present IL-15 in a time frame and quantities matching DCs and that this cis-presentation was as important as DC trans-presentation to elicit NK cell IFN-γ production (29).

### Role

The key role of IL-15 in NK cell biology is underlined by the complete absence of these cells in mice deficient in components of the IL-15 signaling axis (11, 12). However, the role of IL-15 in NK cell physiology is not limited to development. Indeed, this cytokine controls as well survival of mature NK cells in the periphery (24, 25, 30, 31), an effect that is probably mediated by up-regulation of anti-apoptotic Bcl2 family members and down regulation of apoptotic ones (31, 32). Moreover, resting NK cells are poor effectors and need to be primed beforehand to express their full effector capacity. This priming step is also controlled by IL-15, presented by dendritic cells, or monocytes (33–38). Mechanistically, IL-15 signals NK cells to constitute stocks of the effector proteins GzmB and Perforin, absent from unprimed NK cells (33). IL-15, synergizing with IL-12, is also mandatory for IFN-γ expression by NK cells (29, 34, 35). Finally, IL-15 controls NK cell homeostatic proliferation (25, 36, 39) as well as proliferation induced following bacterial, viral, or fungal infections (21, 35, 40, 41). How IL-15 can mediate such a wide range of effects, some homeostatic (differentiation, survival), and some context-dependent (priming, IFN-γ secretion) is still unresolved. One possibility would be that varying IL-15 concentration triggers different responses on NK cells. In line with this idea, decreasing γc expression levels results in reduction of the peripheral NK cell pool (42, 43). This suggests that maximal expression of this receptor and hence maximal signal transduction is necessary for optimal transduction of the IL-15 signal. A recent study tested this model *in vivo* using mouse strains deficient for IL-15Rα or bearing chimeric IL-15Rα either as transgene or knocked in the IL-15Rα locus (44). This strategy allowed the authors to study NK cell populations exposed to five different levels of IL-15 trans-presentation (from null to normal levels). This disclosed the fact that on one hand, constituting a normal peripheral NK cell pool, relying on high proliferation rate in the BM, requires a high level of IL-15 trans-presentation. On the other hand, maturation is much less demanding. The impact of these different levels of IL-15 on the different signaling pathways downstream of the IL-15R has not been analyzed.

### Regulation

How is IL-15 regulated at the basal state remains largely unknown. IRF1, a transcription factor involved in type I IFN (IFN I)-induced IL-15 production, probably plays a role in this process. Indeed, expression of this factor is necessary on hematopoietic as well as non-hematopoietic cells for NK cell generation (45). IL-15 mRNA is expressed *in vivo* by a number of tissues and cell types, from hematopoietic (radiosensitive in chimera experiments) and non-hematopoietic origin (radio-resistant) (24, 46, 47). Chimera experiments have suggested that IL-15 trans-presentation by cells of the hematopoietic system is the most efficient since limiting IL-15Rα expression to the hematopoietic system is sufficient to generate normal NK cell numbers in the BM and only slightly decreased numbers in the periphery (26, 39). In line with its dual function in NK cell homeostasis and activation, IL-15 is expressed at low level under homeostatic conditions in monocytes/macrophages but this expression can be considerably enhanced by several pro-inflammatory agents like LPS (48), poly(I:C), or IFN I (49). More recently, using a transgenic mouse line in which emerald GFP (EmGFP) is expressed under the control of endogenous *Il15* regulatory elements, Lefrançois and collaborators have tracked the cell subsets expressing IL-15 mRNA under homeostatic or inflammatory conditions (50, 51). They confirmed the expression of this cytokine mRNA by a broad distribution of myeloid cells including monocytes, neutrophils, eosinophils, mast cells, and dendritic cells, the strongest expression being observed in basophils. More surprisingly, they described high transcription of IL-15 by Hematopoietic Stem Cells (HSC) and its progressive down regulation during T cell differentiation (51). The significance of this last result awaits further confirmation and functional tests. In addition, IL-15 expression is regulated at several steps including the post-transcriptional level. How much of this regulation is conserved in this reporter remains to be tested. It is however worth noting that these results perfectly correlate with the transcriptomic data available at the Immgen Consortium website (www.immgen.org) for the cell types analyzed (52).

### Signaling

**Figure 2:**
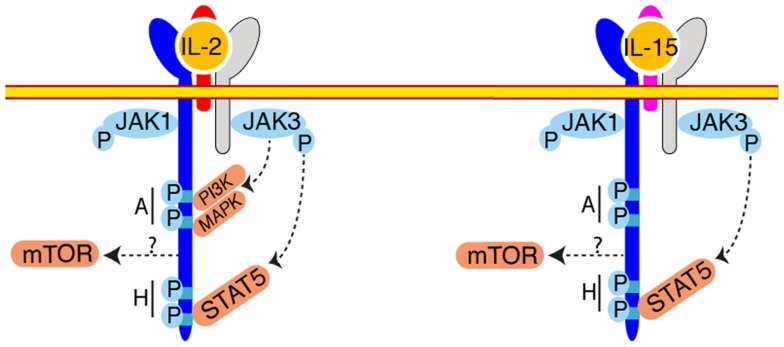
**Signaling downstream IL-2/IL-15Rβ**. Upon engagement of IL-2 or IL-15 different tyrosine residues are phosphorylated in the IL-2/15Rβ intracytoplasmic regions, across two main regions (“A” and “H”). These phosphorylations lead to the recruitment and downstream phosphorylation of STAT5 by JAK1 and JAK3. The mTOR pathway takes also part to IL-15 signaling but the mode of mTOR activation is currently unknown. Activation of mTOR is essential to increase NK cell metabolism. PI3K and MAPK are also activated upon IL-2 engagement and probably also upon IL-15 engagement but this requires formal demonstration.

In terms of signaling, most of our knowledge was generated by studies focused on the IL-2-IL-2 receptor interaction (Figure 2). Given the shared receptor and the similarity of effect of IL-2 and IL-15 on cultured cells, it was inferred that IL-15 stimulation would lead to activation of the same pathways. And indeed, most of the experiments conducted so far suggested a remarkable conservation. However, these two cytokines are not functionally redundant as exemplified by the divergent immunological outcomes of IL-2 or IL-15 treatment (53). A recent *in vitro* study aiming at understanding these differences evidenced subtle changes in the gene transcription induced in CD8 T cells stimulated with IL-2 or IL-15 (54). This observation opens up the possibility that some differences exist in the signaling pathways downstream of the IL-2 or IL-15 receptors. In this context, the exact contribution of the different signaling pathways during NK cell development and activation is poorly understood. Upon IL-2 binding to its receptor, signaling is triggered by Janus Kinases (Jak) 1 and 3, bound to IL-15Rβ and γc (55–58). These kinases phosphorylate tyrosine residues of IL-15Rβ, which serve as docking sites for phosphotyrosine binding proteins such as the Shc adapter protein, Insulin Receptor Substrate (IRS) proteins, and STAT5a and b transcription factors and lead to the activation of three main transduction pathways: the Jak-STAT pathway, the phosphoinositide 3-kinase (PI3K)/Akt pathway, and the Mitogen Activated Protein Kinase (MAPK) pathway. Given its very proximal role in signal transduction, deficiency in Jak3 results in the absence of NK cells (59). In an attempt to dissect the importance of the different pathways stemming from the IL-2/15Rβ subunit, complementation of *Il2/15r*β^−/−^ mice with IL-2/15Rβ transgenes deleted for different cytoplasmic domains was undertaken (60). This approach demonstrated the necessity of the membrane distal H-region (Figure 2) containing tyrosine 392 and 510 and known to recruit STAT3 and 5 and potentially the p85 subunit of PI3K for the generation of a normal NK cell pool (60, 61). In contrast, the truncated protein generated after deletion of the membrane proximal A-region, known to interact with Lck, the p85 subunit of PI3K and Shc, was able to perfectly complement the IL-2/15Rβ deficient mice and to restore NK cell homeostasis and functional response to IL-15 and seems thus dispensable. The importance of the STAT5 pathway was confirmed by several studies (62, 63). Indeed, NK cell number was reduced in STAT5b and to a lesser extend in STAT5a deficient animals and this was associated with a decreased response to IL-2 and IL-15 (63). NK cell specific deletion of both STAT5 factors lead to the complete disappearance of this population resulting from survival defects, probably associated with a block of differentiation at the NKP stage (62). In accordance with the putative role of the H-region of IL-2/15Rβ to recruit p85 via phosphorylated Y392 is a series of studies dissecting the role of the PI3K pathway in NK cell development and functions (64–66). Class IA PI3Ks comprise a p110 catalytic subunit associated with a p85, p55, or p50 regulatory subunit. The p110 catalytic subunits are encoded by three genes *Pik3ca, Pik3cb*, and *Pik3cd*. Class IB PI3Ks consist of the catalytic subunit p110γ (encoded by the *Pik3cg*) associated with the regulatory subunits p101 or p84. NK cells express all catalytic p110 (66), however only the role of p110γ and δ were examined using mice deficient for these proteins (65, 66) or bearing a catalytically inactive form of p110δ (64). Defect in p110γ or δ signaling lead to a decrease in peripheral NK cell number (64–66), combined defect in p110γ∕δ prevented terminal NK cell maturation (66). Moreover, defect in PI3K signaling lead to impaired proliferative (64–66) and cytotoxic (64, 66) responses to IL-2. Importantly, PI3K signaling can be triggered by diverse stimuli including cytokines other than IL-2 or IL-15 (67) chemokines (68) and NK activating receptors (69). The phenotype described in the PI3K deficient NK cells can thus be the consequence of the impairment of responses to other stimuli and not only IL-15. Moreover, direct activation of PI3K by IL-15 was not assessed in these studies neither *in vitro* nor *in vivo* leaving the question of a direct effect unanswered. The generation of phosphatidylinositol triphosphate by PI3K recruits a vast number of targets to the plasma membrane and leads to their activation. However, the downstream targets important for NK cell differentiation and activation have not been investigated. We recently discovered that the kinase mechanistic Target Of Rapamycin (mTOR), which can be activated downstream of PI3K, is a key signaling node activated by IL-15 and responsible for NK cell maturation and activation by pro-inflammatory signals (Marçais et al. manuscript in preparation). mTOR is an evolutionarily conserved serine/threonine kinase integrating various extracellular cues: metabolite and growth factors but also antigenic and inflammatory signals as recently described for T cells (70). mTOR takes part in two complexes: mTORC1 and mTORC2 differing by their constituting members and the targets they phosphorylate. We followed phosphorylation of key mTOR targets by flow cytometry and showed that mTOR activity is developmentally regulated with a progressive shut down upon differentiation and BM egress. In contrast, mTOR activity is strongly induced when NK cells are exposed to pro-inflammatory signals triggered by poly(I:C) injection. Of note, mTOR activation necessitated high IL-15 concentrations; instead, STAT5 phosphorylation was readily triggered by low doses of IL-15. This could provide a first molecular basis to explain the dual effect of IL-15 on NK cells. We also showed that IL-15 controls mTOR activity both *in vitro* and *in vivo*. This is confirmed by the observation that NK cells harvested from mice with NK cell specific mTOR deletion are arrested at the immature CD11b^low^ CD27^high^ stage and their activation in response to poly(I:C) or IL-15 is severely impaired. Interestingly, survival of mTOR deficient NK cells is not affected in accordance with previous studies suggesting that the pro-survival signals given by IL-15 are mediated by STAT5 (62). Preliminary results suggest that only a fraction of mTOR activity is controlled via PI3K. This would fit with the fact that the phenotype of mTOR deficient NK cells is much stronger than PI3K deficient cells. The identity of the relevant mTORC1 or mTORC2 downstream targets remains to be addressed.

Apart from STAT5, the transcription factors, downstream of IL-15 are not characterized. It is worth mentioning the fact that upon MCMV infection, NK cells activate the E2F pathway, a phenomenon that can be blocked using blocking IL-2/15Rβ antibodies (71). As IL-2 has been shown to mediate its proliferative effects through E2F activation (72), we can hypothesize that E2F is also involved in IL-15 induced proliferation. This has however not been formally tested. Moreover IL-15 stimulation leads to NF-κB p65-mediated increase in Myc expression in a context of IL-15 driven leukemia (28).

## IL-2

As discussed above, a number of IL-15 effects are recapitulated by *in vitro* treatment with IL-2. In particular, it has long been known that activated cytotoxic NK cells from BM culture can be generated after exposure to IL-2 (73, 74). However, when IL-2 and IL-15 were compared, 10 to 50 times more IL-2 than IL-15 was needed to activate NK cells (37). This is due to the fact that NK cells do not express the high affinity IL-2 receptor due to their lack of IL-2Rα expression at steady-state. Instead, trans-presentation of IL-15 allows sensing of nanomolar quantities by cells expressing only IL-2/15Rβ and γc (54). Whether IL-2 *in vitro* effects are relevant *in vivo* is difficult to evaluate. Indeed, effect on NK cells of mutations affecting IL-2 signaling are difficult to interpret since they result in overt auto-immunity due to the role of IL-2 in the generation and maintenance of Treg cells. To avoid this caveat, mice deficient both for IL-2 and T cells have been generated. NK cell differentiation and numbers are normal in these *Rag*^−/−^ *Il2*^−/−^ double deficient mice. IL-2 is thus not needed for maintenance of NK cell homeostasis in the absence of T and B cells (13). This is concordant with the fact that, unlike IL-15, which is produced under homeostatic conditions, IL-2 production mainly results from stimulation of the immune system. This cytokine could nevertheless play a role during NK cell priming following inflammatory challenge. Two studies even suggested that IL-2 could play a non-redundant role in this process (75, 76). Indeed, Granucci et al. described a non-redundant role for IL-2 produced by DCs in the first hours following bacterial challenge (76). In this study, DC-derived IL-2 was important for the induction of IFN-γ secretion by NK cells while induction of cytotoxicity was independent of IL-2. A direct contact between the NK and the DC was needed, suggesting that other molecules were involved. This interaction was functionally relevant for bacterial clearance and anti-tumor response. One caveat of this study is however the use of IL-2 deficient DCs differentiated from BM harvested from IL-2 deficient hosts in which T cell dependent auto-immunity develops. Another study described that, following *Leishmania major* infection, the T cell-derived IL-2 was necessary for the induction of IFN-γ secretion by NK cells (75). These findings are challenged by the fact that NK cell priming does not happen in the absence of IL-15 (29, 34, 35). A possibility to reconcile these studies would be to imagine cooperation between the two cytokines, both being needed. In this context, cytotoxicity induction would be under IL-15 control specifically since IL-2 only impacts IFN-γ production in these models (75, 76). The fact that 2 cytokines signaling through the same IL-2Rβ/γc-STAT5-mTOR axis lead to such dissociated effects could be due to difference in the spatio-temporal availability as well as the mode of delivery of these cytokines to NK cells. In any case, this issue remains open for further investigations.

Interest for the IL-2 dependent control of NK cells has recently been renewed by a series of studies proposing that NK cell activity was kept in check by Treg cells buffering excess IL-2 produced by activated T cells (77, 78). In these studies, the authors show that systemic Treg cell depletion leads to an increase in CD4 T cell derived IL-2. This increased IL-2 availability was correlated to increased NK cell IFN-γ production (78) and cytotoxicity toward missing-self targets (77) and could be abrogated by blocking IL-2 antibody treatment. Similar findings have been reported upon transfer of *in vitro* pre-activated NK cells in an irradiated host (79). Mechanistically, Gasteiger et al. linked this increased responsiveness to a better capacity to generate conjugates with target cells after even very short-term exposure to IL-2. Interestingly, this increased cytotoxic capacity was only evidenced against missing-self targets, IL-2 being unable to increase cytotoxicity toward cells expressing both inhibitory and activating ligands. The same group also described phenotypical changes of the NK cell population with the progressive emergence of a CD127^+^ immature NK cell population following Treg depletion (80). A similar population also accumulated in tumor-bearing or chronically infected animals. This population was able to up-regulate IL-2Rα upon low dose IL-12 stimulation confirming previous findings (81). The authors interpreted this result as an increase in the capacity to sense and use IL-2. However, expression of IL-2Rα also renders cells more sensitive to IL-15 (54), the cytokine involved in the homeostasis of this population is thus debatable.

At this stage, we can conclude that DCs and monocytes probably have a prominent role in NK cell activation, through IL-15 trans-presentation. However, this does not exclude the fact that other closely related cytokines like IL-2 and other cell types like T cells or NK cells themselves can contribute and in some conditions replace the IL-15 priming.

## IL-21

IL-21R was discovered independently by two groups, it is homolog to IL-2Rβ and its ligand, IL-21, homolog to IL-2, IL-4, and IL-15 with the strongest similarity with the latest (82, 83). Upon IL-21 binding, IL-21R pairs with γc and signals through JAK1 and STAT5 (82). IL-21 is expressed by activated CD4 T cells while IL-21R is found on lymphoid cells (83, 84). Its first effects described on human NK cells were a potentiation of differentiation from BM progenitors and activation of mature NK cells (83). These effects were in accordance with further studies showing that IL-21 inhibits IL-15 effects on NK cell proliferation but potentiates IL-15 driven NK cell terminal differentiation, i.e., cytotoxicity and IFN-γ secretion (84–87). Forced expression of IL-21 *in vivo* by hydrodynamic plasmid delivery decreases, in an NK cell dependent manner, the number of lung metastasis obtained after tumor cell lines injection (85). Given that IL-21 boosts T cell proliferation *in vitro*, it was suggested that its production by activated T cells could help shutdown the NK cell response once adaptive immunity was functional (84). However no *in vivo* data came to confirm this hypothesis. IL-21 produced by CD4 T cells is essential to prevent CD8 T cell exhaustion during chronic viral infections (88–90). In addition to direct positive effects on antiviral T cells, IL-21 restricts virus-driven Treg cell expansion and their suppressive effect on CD8 T cells (91). Whether IL-21 also controls the function of NK cells during chronic infections remains to be formally tested even though *ex vivo* treatment of NK cells from HIV-infected patients with IL-21 improves their effector function (92, 93).

## IL-7

IL-7, another member of the γc family of cytokines signaling via STAT5, is well known for its role during early steps of B and T cell development in the BM and thymus respectively (94). The fact that early pre-pro NK cells and immature NK cells express high levels of IL-7Rα (6) is puzzling since NK cell development and acquisition of effector functions is perfectly normal in the absence of IL-7 (13, 95). It should be noted that IL-7Rα is also used in combination with the Cytokine Receptor-like factor 2 to form the thymic stromal lymphopoietin receptor (TSLPR). Whether this cytokine plays a role in early stages of NK cell physiology is unknown. It has however been shown to regulate CD8 T cell viability (96). A peculiar thymic IL-7-dependent NK cell subset has also been described (97). This subset is present in minute amount in mice thymi (between 10,000 and 100,000 cells), is virtually absent in *Il7*^−/−^ animals, expresses IL-7Rα and depends on GATA-3 in contrast to BM derived NK cells. Functionally, these NK cells are poorly cytolytic but secrete higher amount of cytokines than conventional NK cells. Given the very low abundance of this population, their function has not been investigated.

## IL-4

IL-4 is also a member of the γc family of cytokines, well known for its pro-Th2 effects during T cell differentiation. Its absence does not affect NK cell generation and homeostasis (13). However, NK cells express the IL-4 receptor as evidenced by their sensitivity to IL-4 treatment *in vitro* (86). Of note is the strong ability of IL-4 to repress some key NK effector functions, such as cytokine production or cytotoxicity. Indeed, it has been demonstrated that IL-4 suppresses the inflammatory cytokine (IFN-γ, TNFα, and GM-CSF) production-increase that is induced following IL-12 treatment in human NK cells (98). Similarly, in mouse NK cells, IL-4 treatment induces a decrease in the cytokine-induced-cytolytic-activity toward tumor cells or immature DC. The mode of action of IL-4 did not involve a down regulation of perforin or granzyme-B by NK cells but could be mediated through NKG2D down regulation (86, 98). In line with these observations, the capacity of NK cells to shape the adaptive immune response and favor a polarized Th1 response through interactions with DCs is abrogated when NK cells are pretreated with IL-4 (99). IL-4 treated NK cells are unable to induce DC maturation and favor tolerogenic or Th2 responses (99).

As mentioned above, another measurable effect of IL-4 was to down regulate NKG2D and other NK cell markers expression *in vitro* and *in vivo* and as a result to decrease NKG2D dependent cell killing (86). Similarly IL-4 treatment has been shown to down regulate NKG2D and CCL5 expression by memory CD8 T cells (100–102). This is in contrast to the promotion, by IL-4, of innate memory-like CD8 T cells generation that has been recently described (103). In the mouse strain Balb/c, high frequency of IL-4-secreting PLZF^+^ NKT cells is associated with increased proportion of memory phenotype CD8 T cells compared to C57Bl/6 mice (104). The generation of this memory population has been shown to be dependent on IL-4, as revealed by the lack of memory phenotype CD8 T cells in *Cd1d*^−/−^ and *Il4r*^−/−^ mice. IL-4 produced during the course of a Th2 response is thought to act in a similar way, as it induces a strong proliferation of memory phenotype as well as naive CD8 T cells (105). The stimulation of memory CD8 T cells by IL-4 induces a strong up-regulation of the Eomes transcription factor (104). Given the implication of Eomes in NK cell differentiation, it is tempting to speculate that IL-4 could have far reaching effects on NK cell biology.

## IL-12 Family

The IL-12 family of cytokines is constituted by heterodimeric cytokines presenting a four α-helix bundle structure and belonging to the IL-6 super-family (106). The constituting heterodimers of this family are formed by combination of two possible β-chains and three possible α-chains. Indeed, the cytokine β-chains are components of two cytokines (p40 of IL-12 and IL-23 and Ebi3 of IL-27 and IL-35) while α-chains include p35, components of IL-12 and IL-35, p19 component of IL-23, and p28 component of IL-27. On the receptor side, IL-12 is recognized by an IL-12Rβ1/β2 receptor, IL-23 by an IL-12Rβ1/IL-23R heterodimer, IL-27 by an IL-27R/gp130 complex, and IL-35 by a gp130/IL-12Rβ2. They have activating as well as inhibitory roles on the immune system, IL-12 and IL-23 being seen as more pro-inflammatory while IL-27 and IL-35 have been more described as inhibitory. Moreover, given the receptors and ligands promiscuity, some members can compete with others generating a complexity far from being understood.

## IL-12

### Discovery/receptors

IL-12 was purified at the end of the 80s from the supernatant of EBV immortalized B cell lines and named NK cell stimulating factor (NKSF) for its ability to induce IFN-γ, cytotoxic activity, and proliferation of NK cells *in vitro* (107). NKSF was later renamed IL-12 and is constituted of two polypeptides: IL-12p40 and p35 covalently linked by disulfide bonds, and binding to a heterodimeric receptor composed of IL-12Rβ1 and β2. Importantly, NK cell constitutively express both chains of the IL-12R (108).

### Regulation/production

IL-12 is produced by several types of Antigen Presenting Cells, including DCs (109, 110) and activated macrophages (48, 111). *In vivo*, IL-12 is produced early after viral infection (112). The inducing signals include pathogen derivative (113), sensed by TLRs and the RIG I pathway, but also molecules expressed by activated T cells like CD40L (109), or the NK cell derived IFN-γ (114–116). The exact mechanism leading to IL-12 production can be more complex as is the case following CpG stimulation. Indeed, upon CpG injection *in vivo*, IL-12 is induced by IL-15 after a cross-talk between conventional DCs and plasmacytoid DCs (117). Interestingly, IL-12 delivery seems to involve the formation of a synapse between the NK cell and the DC involving the polarization of the DC’s secretory apparatus toward the NK cell (118).

### Signaling

Upon binding, IL-12Rβ2 becomes tyrosine phosphorylated and provides docking sites for the kinases tyk2 and Jak2 leading to the phosphorylation and activation of STAT4 (119–121). The importance of tyk2 and STAT4 in NK cells is manifested by the sharp decrease of IFN-γ production in mice deficient for these molecules (40, 121). One report also suggests that some of IL-12 effects are mediated by PKCθ (122), however no further study has confirmed this point.

### Role

The major role of IL-12 *in vivo* is to induce IFN-γ production, while a marginal effect was described at first on early proliferation and development of cytotoxicity (40, 112, 123). Indeed, IFN-γ production is reduced 20-fold in IL-12p35 deficient mice while cytotoxicity is intact (40), antibody mediated blocking of the cytokine leads to similar results (112). Moreover, microscopy studies have shown a perfect time- and location-dependent correlation between IL-12 production by DCs and IFN-γ production by NK cells in *Listeria* induced granuloma (124). As IFN-γ can itself promote IL-12p40 expression, it generates a positive feedback loop promoting inflammation and the differentiation of monocytes into DCs (114, 124). More recently, it has been shown that IL-12 in combination with another cytokine, IL-18, helps optimal NK cell expansion (125, 126) an effect which is not dependent on IFN-γ secretion. Moreover, in some settings, IL-12/IL-18 can drive an IL-15 independent response of NK cells (127, 128). However these results are controversial, indeed, another study found no or only a very minor role of IL-18 and IL-12 for the promotion of the expansion of Ly49H^+^ NK cells during MCMV infection *in vivo* (129). Moreover, the settings used in one study are very peculiar since they involve adoptive transfer of WT NK cells in *Il15*^−/−^ × *Il15r*α^−/−^mice to unmask the IL-12 dependent proliferative effects (128).

**Figure 3:**
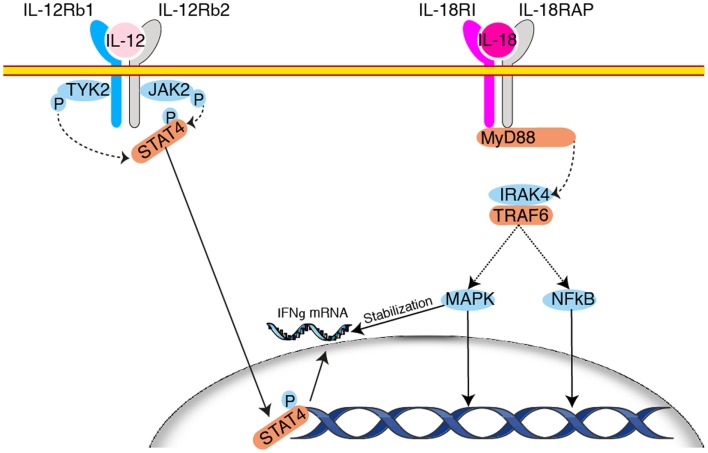
**Synergy between IL-12 and IL-18 for the induction of IFN-γ production**. Engagement of the heterodimeric IL-12 receptor leads to STAT4 phosphorylation after recruitment of the kinases Tyk2 and JAK2. STAT4 transctivates IFN-γ transcription. Upon binding of IL-18 to its receptors there is activation of the MAPK pathway downstream the adapter MyD88. This leads to the stabilization of IFN-γ mRNA and enhances IFN-γ secretion by NK cells. Other mechanisms may also contribute to the synergy between IL-12 and IL-18.

At the molecular level, induction of IFN-γ by IL-12 secretion relies on increased transcription as determined by run-on experiments (130). This transcriptional response is abrogated in tyk2 deficient mice (121). IFN-γ mRNA, like many other cytokine messengers, bears AU rich elements in its 3′UTR, which renders it unstable (131) moreover, it is also a target for miRNA mediated regulation (132). Hence, a large part of its expression relies on post-transcriptional regulation. In this context, it has been shown that IL-18 stabilizes the IFN-γ mRNA through the activation of a MAPK p38 dependent pathway (133) (Figure 3). This effect explains at least in part the formidable synergy existing between IL-12 and IL-18 on induction of IFN-γ secretion (134). Interestingly, IL-12 in combination with IL-18 can also trigger IFN-γ secretion by memory T cells *in vivo* in an antigen independent fashion underlying acquisition of “innate-like” capacities by these cells (38, 135). Conversely, several groups have been able to induce long-term survival of an NK cell population by transferring NK cells briefly activated *in vitro* in the presence of IL-12 and IL-18 in sublethally irradiated (79) or *Rag*^−/−^ hosts (136). This echoes the concept of NK cell memory proposed in 2006 by the group of von Andrian (137). Indeed, under particular circumstances, a fraction of the activated NK pool survives the resolution of the response and is able to mount recall responses (126). This property is severely impaired in the absence of IL-12 (126). How exposure to these cytokines imprints long-term survival onto NK cells in these particular settings is not understood and could constitute fertile ground for further discoveries.

As previously mentioned, IL-12, in synergy with IL-18, has the capacity to induce IL-2Rα on NK cells (81, 86), the CD127^+^ population of NK cells being extremely sensitive to this stimulation (80). This property may help to explain the sustained IL-2-dependent proliferation of IL-15/IL-12/IL-18 pre-activated NK cells after *in vivo* transfer (79). IL-18 is not the only cytokine synergizing with IL-12. Indeed, early studies described a synergy between IL-12 and IL-2 (107). Similarly to IL-18, the synergy between IL-12 and IL-2 also involves regulation of IFN-γ mRNA half-life (130).

## IL-18

IL-18 is a cytokine that was originally identified as an IFN-γ inducible factor (IGIF) (138) and it appears to share its biologic functions with IL-12, including enhancement of the NK cell activity (139). Its absence leads to decreased NK cell response in a variety of models (140, 141). It is part of the IL-1 family which comprises 11 members (142). It is produced as an inactive pro-IL-18 precursor. In contrast to other IL-1 family member such as IL-1β, proIL-18 is constitutively expressed (143). ProIL-18 requires cleavage by active caspase-1 in the inflammasome complex to generate biologically active IL-18 (144, 145). Other proteases [caspase-8 (146, 147), proteinase-3 (148), granzyme-B (149)] have been reported to cleave proIL-18 and generate bioactive cytokine. The physiologic producers of IL-18 include myeloid cells such as activated macrophages (138, 150), dendritic cells (151), neutrophils (152), and Ly6C^+^CCR2^+^ inflammatory monocytes (38). In addition, IL-18 is expressed in numerous non-hematopoietic lineages (153). IL-18 mature form is a leaderless protein secreted via a poorly understood mechanism (154). The ability of IL-18 released by macrophages or DCs to activate the production of IFN-γ by NK cells is dependent on cell-to-cell contact (155–159). In agreement with this cell-to-cell contact requirement, IL-18 secretion by DC is polarized and occurs at the immunological synapse formed between the DC and the NK cell (160). Furthermore, another regulatory step may include the presence of a membrane-bound form of IL-18 as an intermediate between the cytosolic pro IL-18 and the mature soluble IL-18 (161). Finally, IL-18 signaling can be antagonized by IL-18BP, which is limiting the systemic effects of this cytokine (162).

Upon IL-18 binding, its primary receptor, IL-18R1, dimerizes with a second receptor subunit: IL-18R accessory protein (IL-18RAP). This recruits myeloid differentiation primary response protein 88 (MyD88) and initiates signaling through IL-1R-associated kinase 4 (IRAK4) and TNFR-associated factor 6 (TRAF6), leading to activation of the NF-κB and MAPK pathways. The importance of IL-18 for NK cells is underlined by the fact that at steady state, NK cells are the only hematopoietic cells analyzed by the Immunological Genome Project to contain consequent levels of transcripts for IL-18R1 and IL-18RAP (www.immgen.org). This expression grants them with exquisite sensitivity to IL-18 stimulation (150). Moreover, NK from IL-18R1-deficient mice have decreased IFN-γ secretion and cytotoxic capacities (163). A similar phenotype is observed in IRAK4 deficient NK cells (164). Other pathways may also be activated as suggested by impaired IFN-γ secretion in response to IL-12/18 stimulation in p110γ or δ deficient NK cells (65, 66). IL-18 is critical for IFN-γ production by NK cells during numerous bacterial (165), fungal (166), parasites (127), and viral (167) infections. In addition to regulating IFN-γ production, IL-18 takes part to the priming of NK cells (29, 168), and leads to the acquisition of novel migratory function through up-regulation of CCR7 (155, 169). It has also been shown that IL-18 induces the release of CC chemokine Ligand 3 (CCL3) by NK cells, which in turns recruits inflammatory monocytes in the intestine and contribute to local inflammation (170).

The role of IL-18 in regulating the anti-tumoral activity of NK cells is unclear and might be highly dependent of the other signals received by NK cells concomitantly to IL-18. Indeed IL-18 has been shown to promote tumor immuno-suppression and tumor growth by converting Kit^−^ NK cells into Kit^+^ NK cells. NK cells from this Kit^+^ subset have the potential to lyse DCs leading to a reduction in the tumor immuno-surveillance (171). In contrast, others studies have demonstrated an anti-tumor effect of IL-18 (172, 173) in part through the generation of “helper” NK cells producing CCL3 and CCL4, which results to the local recruitment of DCs and effector CD8 T cells (174).

As detailed above, the main effect of IL-18 on NK cells is to synergize with IL-12 to induce IFN-γ production. However, in some systems, IL-12 can be dispensable while IL-18 is not (29, 175). Importantly, if systemic IFN-γ production depends on IL-12/IL-18 synergy, local IFN-γ production in the liver can be preserved in the absence of IL-18 and be sufficient to allow host survival upon MCMV infection (167).

## Transforming Growth Factor-β

### Production

Transforming growth factor-β is a cytokine with a pivotal role in the regulation of the immune system. TGFβ1 is the predominant TGFβ isoform expressed in the immune system. TGFβ associates non-covalently with the latency-associated protein (LAP), forming a complex called the small latent complex (SLC). The SLC can be secreted as such or in association with latent TGFβ-binding protein (LTBP) as a large latent complex (LLC). TGFβ must be released from the complexes to bind to TGFβ receptors (176). This can be achieved through different mechanisms that remain mostly unclear. TGFβ binding proteins control ligand access but can also act as ligand reservoirs (177). Virtually all cells of the immune system can produce TGFβ. TGFβ is regulated at transcriptional, post-transcriptional, and post-translational levels. It is therefore difficult to precisely determine the sources of active TGFβ during immune responses.

### Signaling

Transforming growth factor-β mediates its biological functions through binding to type I and II transmembrane serine/threonine kinase receptors. TGFβ1 signals mostly via TGFBR1 (type I receptor) and TGFBR2 (type II receptor). TGFBR1 is not necessary for binding to TGFβ but initiates signaling. Two signaling pathways have been described that are dependent or not on smad transcription factors. Receptor-associated smads (mostly R-smad 2 and 3 in the immune system) are sequestered in the cytoplasm in the absence of signaling. Upon phosphorylation by TGFBR1, R-smad 2 and 3 interact with the common mediator smad-4 and are translocated into the nucleus. Smad complexes recruit other transcription factors to activate or repress the expression of a wide range of genes. Various Smad-independent TGFβ signaling pathways operate in a context-dependent manner and contribute to cell-specific biological responses. TGFβ may thus activate small GTPases, MAP kinases, and the PI3K pathway (177). In T cells, a JNK-c-Jun pathway has been shown to suppress the expression of Eomes in Th17 cells in response to TGFβ (178). In NK cells, smad-independent TGFβ signaling pathways have not been addressed.

### Impact of TGFβ on NK cells

The addition of recombinant TGFβ in cultures of mouse spleen cells or human PBMC with IL-2 *in vitro* has long been shown to reduce NK cell proliferation and cytotoxicity (179–182). Administration of TGFβ also depresses NK cell activity in mice (183), and reduces their proliferation during antiviral responses (184) while blocking TGFβ increases NK cell cytotoxic activity (185). It was later found that TGFβ also counteracts IL-12 mediated cytokine production by mouse NK cells (186, 187) and human NK cells (188, 189). TGFβ not only counteracts the effects of IL-2 and IL-12 but also reduces IFN-γ production in response to the engagement of the Fc receptor CD16 on human NK cells (190). Finally, TGFβ also shapes the NK cell surface by reducing the expression of NK cell receptors NKG2D and NKp30 (191, 192) and changes their trafficking properties by modulating the expression of chemokine receptors (193).

When does TGFβ act on NK cells *in vivo*? Early studies show that TGFβ is produced during viral infections, especially by T cells at late stages of infection, which could help limiting NK cell cytotoxicity (194). More recent studies show that the transgenic expression of a dominant negative form of the TGFBR2 receptor in CD11c positive cells (including dendritic cells and NK cells) dramatically increases the number of mature NK cells (195), suggesting that TGFβ negatively regulates NK cell development and maturation at steady-state, especially during infancy (196). The smad-dependent pathway has been shown to be important to limit NK cell IFN-γ production by repressing the expression of T-bet (189). Whether this pathway also limits NK cell proliferation and cytotoxicity induced by pro-inflammatory cytokines remains to be determined. Early studies have suggested that TGFβ acts very rapidly, perhaps in a smad3 independent manner to decrease tyrosine phosphorylation induced by IL-2 (197).

## IL-10

IL-10 was described as a Th2 cytokine that inhibited Th1 cytokine synthesis (198). It is now known to be produced by macrophages, DCs, B cells, various subsets of T cells, and NK cells themselves (199–202). NK cells constitutively express both chains of IL-10 receptor (Immgen data). Several diverging effects of IL-10 on NK cells have been described (203–207). Most of these effects seem to be indirect, indeed, IL-10 *in vitro* treatment of purified NK cells does not have noticeable effects (86). However, to the best of our knowledge, this has not been thoroughly tested using chimeras or transfer of IL-10R deficient or sufficient NK cells in IL-10 sufficient hosts. The experiments of *in vivo* blockade using antibodies being non-informative about the responding cell type (208, 209).

## IFN I

IFN-α/β or type I IFN (IFN I) were originally identified as proteins responsible for induction of cellular resistance to viral infections. They are produced by various immune and non-immune cell types. According to a recent study, the capacity of mononuclear phagocytes (i.e., DCs and macrophages) to express IFN I and subsequently IL-15 after microbial challenge could be imprinted by previous contact with the microbial flora (210). However this results still awaits confirmation. IFN I effects on NK cells have been known for a long time. Indeed, IFN I induce NK cell proliferation and cytotoxicity (211). However, IFN I receptor (IFNAR) deficiency can be compensated by recombinant IL-15 injection (40). The bulk of IFN I effects are thus probably mediated through release of IL-15 as recently confirmed by a systems biology analysis of the response to MCMV viral infection (71). In this study, the authors show that IFN-stimulated genes were not strongly up regulated by NK cells, suggesting that, at least at the transcriptional level, these cells were not the primary targets of IFN I. This lower responsiveness was associated with a lower expression of STAT1 by NK cells. IFN I dependent effects on NK cells are however not absent and some studies have evidenced a direct role of IFN I on the induction of NK cell cytotoxicity (212, 213).

## Concluding Remarks

Natural killer cell development and function depend on a multiplicity of cytokines which have complementary as well as overlapping functions. A complete understanding of their action will require the precise identification of the cell types producing them, the time window during which they are produced and the signaling events that their receptors engage in NK cells. These various parameters and the outcome on NK cells could be very different depending on the infectious agent. The role of some cytokines such as IL-2 may thus be important only with particular pathogens and efforts should therefore be made to diversify the models of infection. Moreover, many cytokine effects are only seen when combining them, as best exemplified by IL-12 and IL-18. It is therefore essential to define the relevant cytokine combinations in different niches and to delineate the signaling pathways they induce as well as their combined effects on NK cells. How NK cells integrate signals from activating and inhibitory cytokines and which molecules act as “integrators” are important issues to address. Recent technological advances such as mass cytometry will be instrumental for this purpose in that they allow the simultaneous measurement of up to 100 parameters using very low cell numbers. The latter technique can be applied to the study of cell signaling using phospho-specific antibodies raised against various molecules of the transduction machinery. Recently generated NK-specific Cre-expressing mouse lines will also be important to discriminate between direct vs. indirect effects of various cytokines on *in vivo* NK cell physiology.

## Conflict of Interest Statement

The authors declare that the research was conducted in the absence of any commercial or financial relationships that could be construed as a potential conflict of interest.
